# Phenolic Content and Antioxidant Activity in Seeds of Common Bean (*Phaseolus vulgaris* L.)

**DOI:** 10.3390/foods10040864

**Published:** 2021-04-15

**Authors:** Roberto Rodríguez Madrera, Ana Campa Negrillo, Belén Suárez Valles, Juan José Ferreira Fernández

**Affiliations:** 1 Área de Tecnología de los Alimentos, Regional Agrifood Research and Development Service (SERIDA), E-33300 Villaviciosa, Asturias, Spain; mbsuarez@serida.org; 2 Área de Cultivos Hortofrutícolas y Forestales, Regional Agrifood Research and Development Service (SERIDA), E-33300 Villaviciosa, Asturias, Spain; acampa@serida.org (A.C.N.); jjferreira@serida.org (J.J.F.F.)

**Keywords:** phenolic content, flavonoids, anthocyanin, antioxidant activity, DPPH, FRAP, common bean, *Phaseolus vulgaris* L.

## Abstract

Dry bean (*Phaseolus vulgaris* L.) is one of the most important pulses consumed in the world. Total phenolic content, total flavonoid content, total monomeric anthocyanin content and antioxidant capacity were determined, using ferric reducing antioxidant power and free radical scavenging activity, in 255 lines grown under the same environmental conditions. For all parameters analysed, there was a wide range of variability, with differences always above one order of magnitude. Phenolic compounds in beans with coloured coats were found to be more efficient antioxidants than those with completely white coats, and samples with more strongly coloured coats (red, cream, black, pink and brown) showed the highest antioxidant capacities. Based on the strong correlation detected between the variables, total phenolic content can be considered an appropriate indicator of antioxidant activity. The results provide a robust database for selecting those lines of greater functional and nutritional interest in terms of cultivation for direct consumption, for inclusions in food formulations or for use in future breeding programs.

## 1. Introduction

Common bean (*Phaseolus vulgaris* L.) is one of the most important pulses cultivated and consumed the world over, with an estimated annual production of around 12 million tonnes [1]. Among the reasons for the success of this crop is its nutritional interest, with its high content of protein, dietary fibre, complex carbohydrates, vitamins, minerals and phytochemicals, which exert protective effects against various diseases [2,3].

Phenolic compounds are one of the most important families of phytochemicals present in beans. These molecules play an important role in human health because they possess antioxidant activity related to anti-diabetic, anti-obesity, anti-inflammatory, anti-mutagenic and anti-carcinogenic properties [4].

The phenolic composition of *P. vulgaris* seeds has been described in several works, showing a clear difference between the compounds constituting cotyledons and coat. Specifically, derivatives of phenolic acids are major compounds in cotyledons (mainly *p*-coumaric, ferulic and cinnamic acids), while in the coat, there are different flavonoids and, to a lesser extent, tannins [5,6,7,8,9,10,11].

It has been shown by various authors that differences in phenolic composition between varieties could be related to the colour of the seed. However, detailed studies suggest that variability in phenolic content is due more to genotype than seed coat colour [5,10]. In addition, environmental conditions can also affect the phenolic content of dried beans [12,13].

The information reported on antioxidant activity in dry bean is usually in reference to limited diversity with few and selected varieties or cultivars being examined. In addition, antioxidant activity is estimated following different methods and the phenolic compounds are extracted by a variety of procedures and it is therefore difficult, and sometimes impossible, to assess the existence, or not, of differences in the antioxidant activity reported for different varieties [12,14,15,16,17,18,19,20,21,22].

Having an appropriate chemical characterisation of the plant material available in a collection that includes the widest possible variability of the species allows, on the one hand, the identification of those genotypes which may be more interesting from a nutritional and functional point of view, and on the other, the establishment of a powerful database for future breeding programs.

*P. vulgaris* is a legume originally domesticated by the pre-Columbian civilisations in two principal geographical areas: the Andean and the Mesoamerican [23,24]. Local bean accessions collected in Spain in the middle of the 20th century include wide phenotypic and genotypic diversity for both gene pools described in this species, as well as materials probably derived from recombination between pools [25]. Most of this genetic diversity is included in the Spanish Diversity Panel (SDP) [26].

Taking into account the interest in having a database that collates the largest possible variation present in the species, the characterisation of the SDP was carried out by establishing the phenolic profile of samples by high-performance liquid chromatography (HPLC), thereby identifying and quantifying 40 phenolic components of dry beans [10].

The objective of this work is to complete the characterisation of the SDP of common bean seeds by determining the samples’ antioxidant activity and total content of phenol, flavonoid and anthocyanin in ethanol extracts.

## 2. Materials and Methods

### 2.1. Plant Material

Two hundred and fifty-five lines of *Phaseolus vulgaris*, all of which are included in the Spanish Diversity Panel (SDP) [26], were used in this study. This panel consists of lines established from local Spanish germplasm, as well as old and elite cultivars mainly used for snap consumption. Most of the landraces included are derived from the Spanish common bean core collection. Lines were grown in a greenhouse at the Regional Agrifood Research and Development Service (SERIDA), Villaviciosa, Asturias, Spain (43°29′01″ N, 5°26′11″ W; elevation 6.5 m) in 2018 using a randomised complete block design with one replicate per line, consisting of ten plants distributed in a 1 m row plot. The dry pods were manually harvested and threshed. The dry seeds were kept under controlled conditions (−20 °C under vacuum) until they were analysed. The lines were classified according to the main seed coat colour [27,28], into white (*n* = 71), white with speckle (*n* = 18), yellow (*n* = 12), cream (*n* = 41), brown (*n* = 34), red (*n* = 20), pink (*n* = 17), grey (*n* = 6) or black (*n* = 36).

### 2.2. Sample Treatment and Extraction

Extraction of polyphenols was carried out according to a previously validated method [29]. Briefly, 50 g of seeds per line were ground in a coffee grinder and passed through a standard sieve (number 18 corresponding to a sieve open ring size of 1.00 mm). The flours (1.5 g) were extracted with 30 mL of 46% aqueous ethanol (0.1% perchloric acid), over a period of 10.3 min, in a water bath at 20 °C using an ultrasonic homogeniser UP200Ht (Hielscher, Teltow, Germany) equipped with a 2 mm diameter sonotrode at a frequency of 26 kHz. After extraction, the solids were separated by centrifugation, the supernatant was dried in a rotary vacuum evaporator at 40 °C, after which the residue was reconstituted with 4 mL of 20% aqueous methanol (0.1% perchloric acid) and filtered through a 0.45 µm polyvinylidene difluoride (PVDF) syringe filter. Two extractions were carried out per sample.

### 2.3. Phenolic Composition

#### 2.3.1. Total Phenolic Content (TPC)

Total phenolic content (TPC) was determined by spectrophotometry using the Folin–Ciocalteu method [30]. The reaction is developed in 10 mL volumetric flasks, to which the various reactants are added in this order: 200 µL of appropriately diluted extract, 5 mL of water, 250 µL of Folin–Ciocalteu reagent, 750 µL of 20% sodium carbonate and water to reach the final volume. The absorbance was measured at 700 nm, after 30 min at room temperature. Gallic acid was used as standard for the quantification of total phenolic compounds. The results are expressed as µg gallic acid equivalent (GAE)/g. All extracts were analysed in triplicate.

#### 2.3.2. Total Flavonoid Content (TFC)

Total flavonoids were measured by the aluminium chloride method according to Kim et al. [31], with slight modifications. A 500 µL aliquot of appropriately diluted extract was added to a 5 mL volumetric flask containing 2 mL distilled water and 150 µL of 5% NaNO_2_. After 5 min, 150 µL of 10% AlCl_3_·6H_2_O was added and mixed and 6 min later, 1 mL 1 M NaOH and 1.2 mL distilled water were added and thoroughly mixed. The absorbance was measured at 510 nm versus a water blank. Catechin was used as standard for the quantification of total flavonoids. Results were expressed as µg catechin equivalent (CE)/g. All extracts were analysed in triplicate.

#### 2.3.3. Monomeric Anthocyanin Content (MAC)

Monomeric anthocyanin content (MAC) was determined by the differential pH method described by Wrolstad [32]. Briefly, two aliquots of 500 µL of appropriately diluted extracts were diluted 1/5. One aliquot was diluted with pH 1.0 buffer (0.025 M potassium chloride) and the other was diluted with pH 4.5 buffer (0.4 M sodium acetate). Absorbance of diluted samples was measured at 510 and 700 nm using a Perkin–Elmer Lambda 35 UV spectrophotometer (Boston, MA, USA). MAC in the extracts was calculated according to the formula:

MAC = [(A _λ500_ − A _λ700_)_pH1_ − (A _λ500_ − A _λ700_)_pH4_._5_] × 449.2 × 1000/(26,900 * DF) (1)

where A is the absorbance, 449.2 is the molecular mass of cyanidin-3-*O*-glucoside, 26,900 is its molar absorptivity (ε) and DF is the dilution factor. The results were expressed as µg cyanidin-3-*O*-glucoside equivalent (C3G)/g. All extracts were analysed in triplicate.

### 2.4. Antioxidant Activity

#### 2.4.1. Reducing Power

Reducing power was carried out by the ferric reducing antioxidant power (FRAP) method, according to Benzie and Strain [33]. Working FRAP reagent was prepared daily from the following three solutions in the ratio 10:1:1: 300 mM acetate, pH 3.6; 10 mM TPZ (2,4,6-tripyridyl-s-triazine) in 40 mM HCl and 20 mM FeCl_3_·6H_2_O. Briefly, 100 µL of appropriately diluted extracts were mixed with 3.0 mL working FRAP reagent in a test tube, and the absorbance was read at 593 nm against a reagent blank after 20 min at room temperature. FeSO_4_·7H_2_O solutions were used to construct a standard curve and the results were expressed as µmol Fe (II)/g. All extracts were analysed in triplicate.

#### 2.4.2. Radical Scavenging Activity

Radical scavenging activity was determined using the 2,2-diphenyl-1-picryl-hydrazyl-hydrate (DPPH) method according to Diñeiro Garcia et al. [34]. Forty µL of either appropriately diluted extract, the standard or methanol in the case of the reagent blank, were added to 1.460 mL of DPPH solution (1 × 10^−4^ M) in methanol. Samples were diluted with methanol to ensure that the readings were in the linear range of the standard curve. Absorbance at 515 nm was measured after 120 min when the reaction reached its stable state. The inhibition percentage (IP) was calculated as follows:

%IP = ((A_blank_ − A_sample_)/A_blank_) × 100 (2)

where A_sample_ is the absorbance of the solution in its stable state and A_blank_ is the absorbance of DPPH solution when methanol is added rather than the sample. Trolox solutions were used to construct a standard curve and the results were expressed as µmol trolox equivalent (TE)/g. All extracts were analysed in triplicate.

#### 2.4.3. Phenol Antioxidant Index

The phenol antioxidant index (PAOXI) is an indicator introduced by Vinson and Hontz [35] which takes into account both the concentration of the antioxidant phenols and their antioxidant effectiveness. PAOXI was calculated according to Pereira and Tavano [36] as follows:

PAOXI = (μmol of DPPH inhibited/g)/(mg GAE/g)(3)

### 2.5. Statistical Analysis

A one-way analysis of variance (ANOVA) was carried out to evaluate the influence of bean coat colour on phenolic content and antioxidant activity. Differences between colour groups were detected by a Duncan’s test for mean comparisons. Pearson’s correlation coefficient (R) was computed to estimate correlations between variables. The program used was SPSS version 15.0 (SPSS Inc., Chicago, IL, USA).

K-means clustering was conducted in R software (R core team 2020) and visualised using the packages Factoextra [37], ggplot2 [38] and ggpubr. Significant differences between the clusters identified were investigated using Duncan’s tests for each variable evaluated.

## 3. Results and Discussion

Detailed information on each line analysed, their phenotypic characteristics, TPC, TFC, MAC, FRAP, DPPH and PAOXI is available as Supplementary Materials (Table S1).

### 3.1. Phenolic Composition

**Figure 1:**
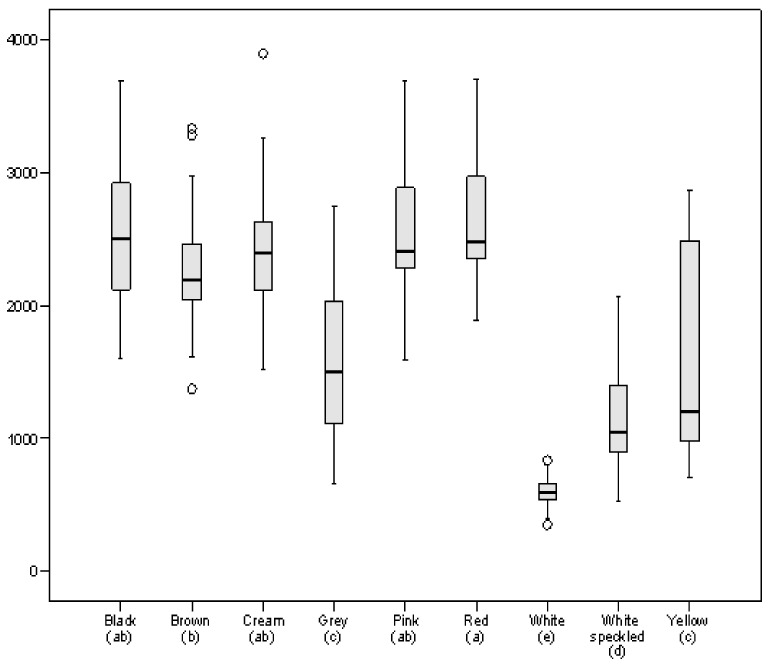
Boxplot representing total phenolic content (TPC), expressed as µg gallic acid equivalent (GAE)/g, in the bean panel grouped by seed coat colour. Different letters in parentheses indicate significant differences at *p* < 0.05.

The panel of samples (255 lines) presented differences of one order of magnitude in total phenolic content (TPC), which demonstrates the variability of the species (Figure 1; Supplementary Table S1). TPC ranged between 350 (line SDP221; colour: white) and 3894 µg GAE/g (SDP061; cream). The white coat lines presented the lowest average TPC content (average: 600 µg GAE/g), compared to a large group formed by lines of different colours which showed average values of between 2238 (brown) and 2624 µg GAE/g (red). These data are in line with those reported by other authors, both for varieties of white seed [39] and coloured seed [17,40,41,42].

**Figure 2:**
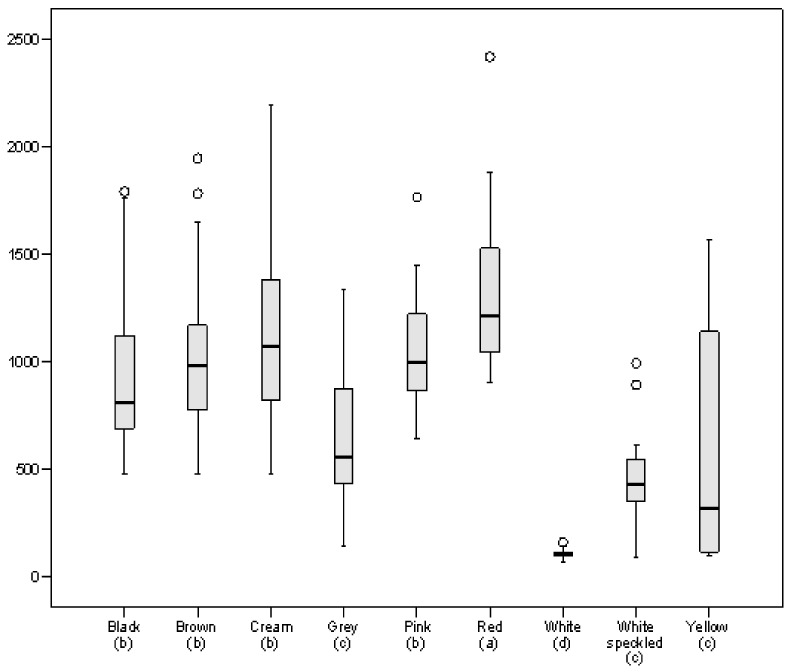
Boxplot representing total flavonoid content (TFC), expressed as µg catechin equivalent (CE)/g, in the bean panel grouped by seed coat colour. Different letters in parentheses indicate significant differences at *p* < 0.05.

The seed coat colour of common beans is due to the presence of various phenolic components, among which flavonoids play an important role [43]. As was expected, the white coat beans contained the lowest levels of flavonoids (Figure 2; Supplementary Table S1), with an average value of 106 µg CE/g, compared to the highest content, which was detected in red coat beans (average: 1325 µg CE/g). Other groups of beans with a high flavonoid content were cream coat lines (average: 1102 µg CE/g), pink (average: 1067 µg CE/g), brown coat (average: 1030 µg CE/g) and black coat (average: 915 µg CE/g), as was also observed for TPC. These results, and the high correlation detected between TPC and TFC in the panel formed by the 255 lines (R_TPC/TFC_ = 0.933, *p* < 0.001), show the important contribution of flavonoids to the total polyphenol content (Supplementary Figure S1). The ranges of variability found here are in accordance with data reported by other authors for varieties of different phenotypes [17,36,40,44].

**Figure 3:**
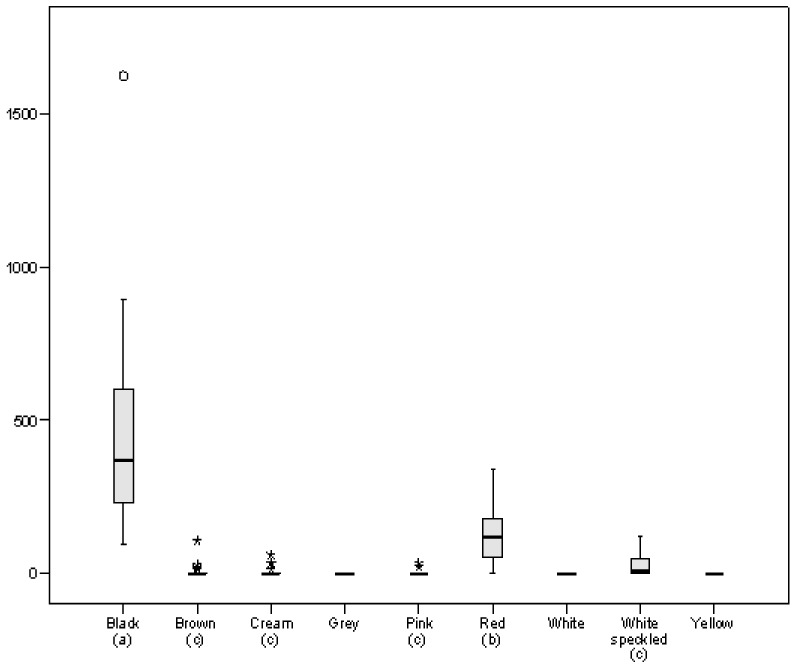
Boxplot representing anthocyanin content (MAC), expressed as µg cyanidin-3-O-glucoside equivalent (C3G)/g, in the bean panel grouped by seed coat colour. Different letters in parentheses indicate significant differences at *p* < 0.05.

Anthocyanins are a type of flavonoid that give red, blue or purple pigmentation to plants. Black coat beans stood out both qualitatively and quantitatively for their MAC content (Figure 3), with all samples in this group showing the presence of this type of flavonoid, and it being the group where the highest anthocyanin content was found (average: 440 µg C3G/g). Red coat beans are also interesting in terms of their anthocyanin content, with the group having an average MAC value of 128 µg C3G/g and anthocyanins being present in 18 of the 20 red samples. The presence of monomeric anthocyanins in white coat beans with red speckles should also be noted, where MAC was present at levels up to 120 µg C3G/g (line SPD185). Individually, the black line SDP097 had the highest MAC (1623 µg C3G/g), almost double that of the line with the next highest value (893 µg C3G/g, SDP277, black). These results are in accordance with data reported by Salinas-Moreno et al. [45] with respect to anthocyanin content in 15 Mexican black bean varieties, which ranged between 388 and 719 µg/g, as well as, in general, with the values reported by Aquino-Bolaños et al. [46] for varieties with different pigmentation, although it should be noted that these latter authors detected the highest content in varieties defined as cream-pink (9070 µg C3G/g).

The fact that similar seed coat colours can result from various possible combinations of compounds means that the colour classification does not guarantee that two beans with similar coloured coats have a homogeneous composition in terms of phenolic compounds. In general, values for TFC and MAC varied far more in the coloured samples than in the totally white samples, as is exemplified by the relative standard deviation (RSD) of 95% in TFC for yellow coat beans and 345% for the MAC content of cream coat beans. In contrast, values for TPC, which encompasses other groups of compounds such as phenolic acids, not just flavonoids, were more homogeneous, with RSD ranging between 15% (white) and 51% (yellow).

As has been shown, the species *P. vulgaris* shows wide diversity in the content of phenolic compounds, with values similar to cultivars of other legumes widely consumed worldwide such as pea, chickpea, lentil and soybean [17,41,47,48,49], which highlights the importance of beans as a source of phenolic compounds.

According to the criteria of Marathe et al. [41] for classifying legumes by their TPC content, 67% of the samples in our panel could be considered as medium or high TPC (>1000 µg GAE/g), with white coat beans being the only lines where TPC was not equal to or greater than this value.

### 3.2. Antioxidant Activity

Table 1 shows the value of antioxidant activity (FRAP and DPPH) and the phenol antioxidant index (PAOXI) for lines grouped according to their seed coat colour. The results of these parameters for each line are shown in Supplementary Table S1.

The FRAP method measures the ability of a sample to reduce the Fe^3+^ in the Fe^3+^-TPTZ complex to Fe^2+^. Here, the FRAP assay showed values for reducing power ranging from 3.68 to 72.93 μmol Fe (II)/g, corresponding to a white line (SDP221) and a black line (SDP097), respectively. The black coat bean group was that with the highest reducing power (42.3 μmol Fe(II)/g), around 7 times higher than white coat beans (6.1 μmol Fe(II)/g), the lowest, while the average for the panel of 255 samples was 23.03 μmol Fe(II)/g (Table 1, Supplementary Table S1). Xu et al. [48] reported values of reducing power in bean extracts using the FRAP method that ranged from 12.7 μmol Fe(II)/g for the Navy bean (white) to 97.0 μmol Fe(II)/g for the Black Turtle Eclipse bean (black), while Orak et al. [21] reported values between 25 and 46 μmol Fe (II)/g in two red beans, which is in line with our results.

The DPPH method measures the ability of compounds to act as scavengers of the 2,2-diphenylpicrylhydrazyl radical. Use of an ANOVA and Duncan′s test of radical scavenging measured by the DPPH method showed that the samples from the SDP could be divided into 5 groups (Table 1). The first two are composed of the 5 coloured categories, which had the highest antiradical capacity, these being, in descending order: red, black, cream, pink and brown groups (average values between 18.7 and 14.8 μmol TE/g). The next two groups had average values of between 8.9 and 5.2 μmol TE/g, i.e., the grey, yellow and white speckled samples, while the final group was comprised of only the totally white coat beans and had the lowest antiradical power of 1.2 μmol TE/g (Table 1). Radical scavenging showed high variability between lines, ranging from 0.7 μmol TE/g for the white line SDP221 and 38.1 μmol TE/g for the cream line SDP192 (Supplementary Table S1). Values between 7.1 and 32.4 μmol TE/g were reported by Aquino-Bolaños et al. [46] in common Mexican bean landraces with coloured coats.

PAOXI values were the indicator associated with antioxidant capacity that showed the lowest variability (Supplementary Table S1), less than one order of magnitude, with values of between 3.4 (SDP273) and 32.1 (SDP192). The interesting point about PAOXI is that it can establish the relationship between antioxidant activity and phenolic composition, which is not always so direct [50]. Ombra et al. [50] noted that although there may be a good correlation between TPC and DPPH, varieties with high TPC content do not always give the highest DPPH values, and these facts could be attributable to differences in individual composition in each case. Comparison of some of the lines in our study also showed that lines with similar TPC content, such as SPD008 (TPC: 3135 µg GAE/g; cream) and SDP047 (3067 µg GAE/g; pink), have very different antiradical scavenging values, in this case 33.23 and 17.2 μmol TE/g respectively, which demonstrates the greater effectiveness as antioxidants of the phenolic compounds present in SPD008 (PAOXI: 28.4) compared to those of SDP047 (PAOXI: 15.1). Likewise, lines with similar DPPH values, such as SPD151 (15.0 μmol TE/g; black) and SDP143 (15.2 μmol TE/g; pink), have TPCs of 1855 μg GAE/g and 2936 μmol TE/g respectively, thus indicating that the phenolic compounds present in SPD151 (PAOXI: 21.75) are more effective as antioxidants compared to those in SDP143 (PAOXI: 13.91). In this sense, it should be pointed out that within a group of beans with the same colour coat and even within the same phenotype (market class), it is possible to find some genotypes with very different phenolic profiles [10], which would justify differences in antioxidant activity at the genotype level. However, these differences, which should be considered as exceptions of great potential interest from a genetic and breeding point of view as they provide diversity to the group, are not detected when the beans are analysed grouped by coat colour. In this case, the groups with the highest values for TPC, TFC, FRAP and DPPH also had the highest PAOXI values, with only slight variations in the order of classification for each parameter that were irrelevant from a quantitative point of view (Table 1).

Significant correlations were detected between TPC and both FRAP and DPPH (R_TPC/FRAP_ = 0.918, *p* < 0.001; R_TPC/DPPH_: 0.956, *p* < 0.001; Supplementary Figures S2 and S3), indicating the suitability of TPC, and to a lesser extent TFC, as an indicator of antioxidant activity (R_TFC/FRAP_ = 0.830, *p* < 0.001; R_TFC/DPPH_: 0.935, *p* < 0.001; Supplementary Figures S4 and S5), thus revealing the contribution of other non-flavonoid phenolic components to the antioxidant activity of the extracts. Correlation between TPC, FRAP and DPPH have also been reported by several authors in *P. vulgaris* [20].

**Figure 4:**
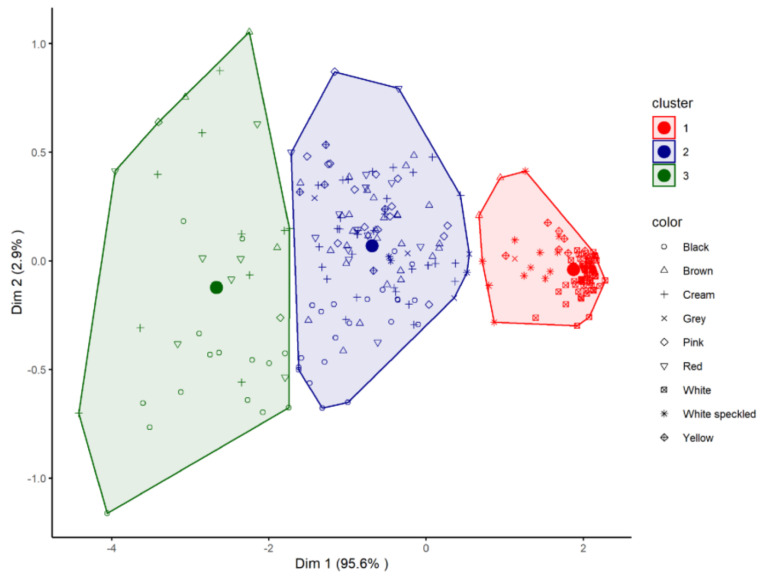
Grouping of *P. vulgaris* lines studied according to their radical scavenging activity, reducing power and total phenolic content by K-means clustering. The seed coat colour of each line is indicated.

In order to detect natural groupings among the samples constituting the database, a cluster analysis was performed with the variables associated with antioxidant activity (TPC, FRAP and DPPH). As can be seen in Figure 4, three clusters that were significantly different from each other were detected.

-Cluster 1, consisting of 97 samples, mostly white or white speckled (*n* = 86) and some samples of different colours (7 yellow, 2 grey and 2 brown). According to Duncan’s test, this group showed the lowest average values in TPC (713 µg GAE/g), FRAP (7.2 µmol Fe (II)/g) and DPPH (1.9 µmol TE/g).

-Cluster 2, comprising 121 samples, which was the most heterogeneous group, with samples of all colours except completely white coat beans. Their phenolic content and antioxidant capacity were intermediate, and statistically different, to that of clusters 1 and 3 (TPC: 2231 µg GAE/g; FRPA: 28.3 µmol Fe (II)/g; DPPH: 13.9 µmol TE/g).

-Cluster 3, formed by 37 samples of black (15), cream (10), red (7), brown (3) and pink (2) beans, and showed the highest average values for the three parameters (TPC: 3071 µg GAE/g; FRAP: 47.3 µmol Fe (II)/g; DPPH: 24.8 µmol TE/g).

As was discussed above with regard to TPC content, Marathe et al. [41] classified legumes according to their antioxidant activity into having low, medium or high antioxidant power. Although these authors established the groups for convenience, the fact is that the statistically significant groups resulting from the Duncan’s test performed in the current work (Figure 1 and Table 1) fit the intervals suggested by these authors reasonably well in terms of TPC and FRAP values. Furthermore, as has been stated, the database shows a natural grouping of the samples into three clusters according to their phenolic content and antioxidant activity (Figure 4).

Consequently, from these groups, and taking into account the correlation detected between TPC and both FRAP and DPPH, we have established a 3-group classification of the samples based on their phenolic and antioxidant power (Supplementary Table S1):

-High phenolic content and antioxidant activity (HPA): >2000 µg GAE/g; >30 µmol Fe (II)/g; >20 µmol TE/g.

-Medium phenolic content and antioxidant activity (MPA): >1000 µg GAE/g; >15 µmol Fe (II)/g; >5 µmol TE/g.

-Low phenolic content and antioxidant activity (LPA): <1000 µg GAE/g; <15 µmol Fe (II)/g; <5 µmol TE/g.

As polyphenol content does not always correlate with reducing power or radical scavenging activity, a sample should only be considered as HPA or MPA if it meets the three criteria of its group. Of the 255 lines analysed, 28 can be considered as HPA, 133 as MPA and 94 as LPA (Supplementary Table S1).

A contingency test between the two grouping variables (clusters from K-means analysis and grouping according to phenolic and antioxidant power) was significant (*p* < 0.001), indicating correspondence between the two systems of classification (cluster 1 = LPA, cluster 2 = MPA and cluster 3 = HPA). This correspondence was successful in more than 95% of the cases; specifically, 121 of the 133 lines defined as MPA were located in cluster 2 (91%) and 100% of LPA and HPA were included in clusters 1 and 3, respectively.

## 4. Conclusions

Individually, notable differences were detected between lines, with ranges of variability being one order of magnitude higher both in terms of phenolic compounds (TPC, TFC and MAC) and antioxidant activity (reducing power and radical scavenging activity). Moreover, the high correlation detected between these parameters shows the suitability of TPC as an indicator of antioxidant activity. In general, it can be concluded that beans with more strongly coloured coats (red, cream, black, pink and brown) are those which are more interesting from the functional point of view, having higher levels of total phenolic compounds (TPC > 2000 µg GAE/g), reducing power (FRAP > 30 µmol Fe (II)/g) and radical scavenging activity (DPPH > 20 µmol TE/g). On the contrary, the completely white coat beans presented the lowest levels of these parameters (TPC < 1000 µg GAE/g; FRAP < 15 µmol Fe (II)/g); DPPH < 5 µmol TE/g). The phenol antioxidant index (PAOXI) showed that, in general, phenolic compounds in beans with coloured coats have a higher efficiency as antioxidants than those in completely white ones. The database generated in this work provides a robust database for the selection of those lines that are of more interest from a functional or nutritional point of view, so that they can be cultivated for direct consumption, including in food formulations or used in future breeding programs.

## Figures and Tables

**Table 1:** Antioxidant activity and phenol antioxidant index in common beans according to their seed coat colour (mean ± standard deviation).

	*n*	Reducing Power ^1^	Radical Scavenging Activity ^2^	PAOXI ^3^
Black	36	42.3 ± 11.3 a	17.4 ± 5.1 ab	18.1 ± 2.7 ab
Brown	34	27 ± 6.6 c	14.8 ± 5.1 b	17.4 ± 3.2 ab
Cream	41	31.4 ± 10.4 bc	17.2 ± 7.0 ab	18.9 ± 4.2 a
Grey	6	19.3 ± 9.2 d	8.9 ± 5.5 c	13.8 ± 4.1 c
Pink	17	27.8 ± 6.8 c	14.9 ± 5.1 b	15.9 ± 2.5 bc
Red	20	34.1 ± 10.4 b	18.7 ± 6.3 a	18.7 ± 3.2 a
White	71	6.1 ± 1.6 f	1.2 ± 0.4 e	5.3 ± 1.4 e
White speckled	18	13 ± 6.7 e	5.2 ± 3.5 d	11.2 ± 3.8 d
Yellow	12	16.8 ± 11.2 de	8.0 ± 7.4 cd	10.3 ± 6.2 d

^1^ Reducing power calculated by the ferric reducing antioxidant power (FRAP) method, values expressed as μmol Fe (II)/g. ^2^ Radical scavenging activity calculated by the 2,2-diphenyl-1-picryl-hydrazyl-hydrate (DPPH) method, values expressed as μmol TE/g. ^3^ PAOXI: phenol antioxidant index, calculated according to Pereira and Tavano [36]. Different letters in a column indicate significant differences at *p* < 0.05. *n*: number of lines.

## Data Availability

Data is contained within the article or supplementary materials.

## Supplementary Materials

The following are available online at https://www.mdpi.com/article/10.3390/foods10040864/s1, Figure S1: Correlation between TPC and TFC, Figure S2: Correlation between TPC and FRAP, Figure S3: Correlation between TPC and DPPH, Figure S4: Correlation between TFC and FRAP, Figure S5: Correlation between TFC and DPPH, Table S1: Total phenolic content, total flavonoid content, monomeric anthocyanin content, reducing power, radical scavenging activity and phenol antioxidant index on each line analysed.
