# Supplementary figures and images for: Phenolic Content and Antioxidant Activity in Seeds of Common Bean (Phaseolus vulgaris L.)
Source: Foods. 2021 Apr 15;10(4):864. doi: 10.3390/foods10040864 (PMC8071416; doi:10.3390/foods10040864)

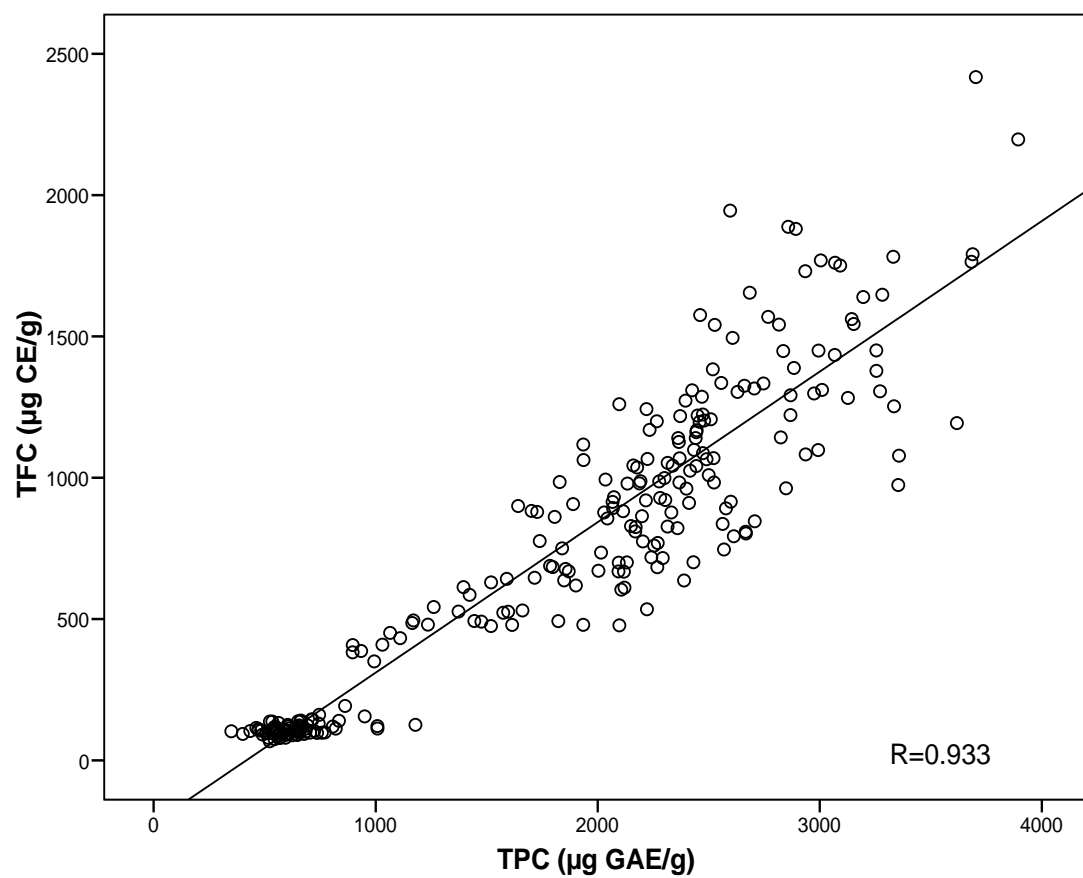

Figure S1. Correlation between TPC and TFC.

Supplement: Supplementary file 1 [file foods-10-00864-s001.zip › Supplementary Figures 1.pdf]

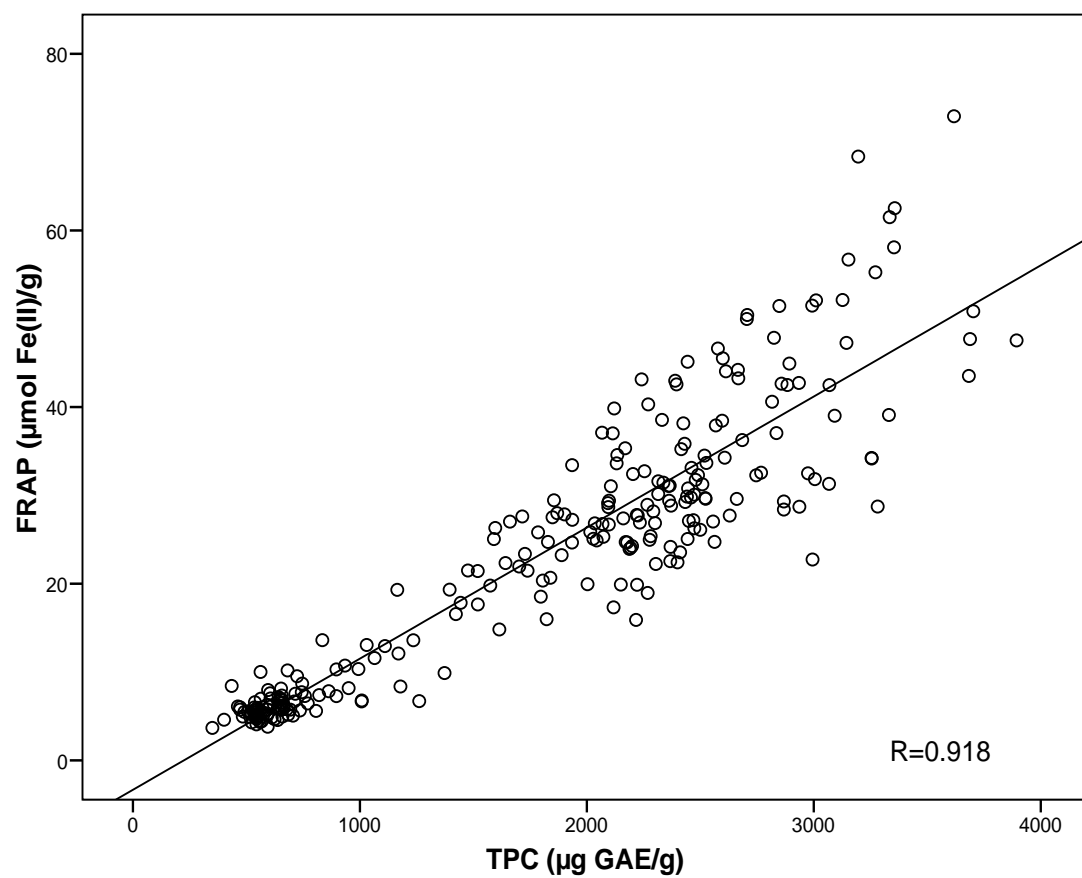

Figure S2. Correlation between TPC and FRAP.

Supplement: Supplementary file 1 [file foods-10-00864-s001.zip › Supplementary Figures 2.pdf]

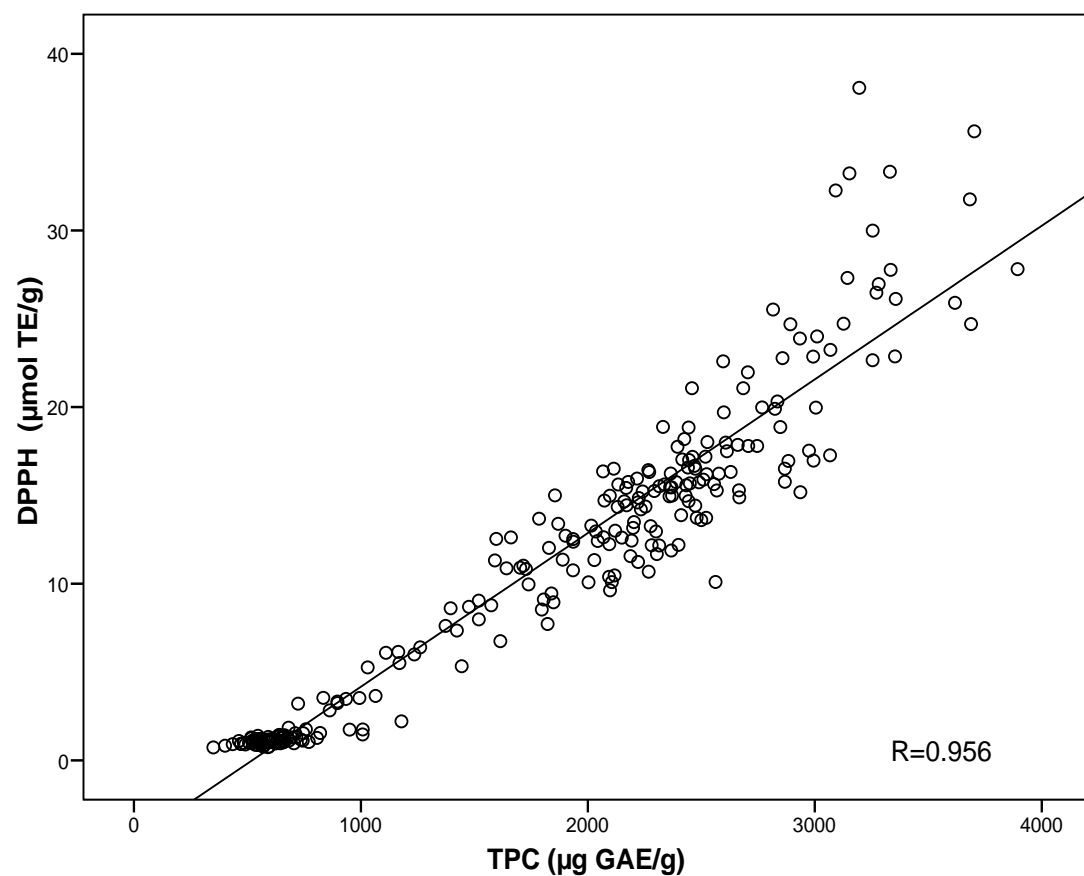

Figure S3. Correlation between TPC and DPPH.

Supplement: Supplementary file 1 [file foods-10-00864-s001.zip › Supplementary Figures 3.pdf]

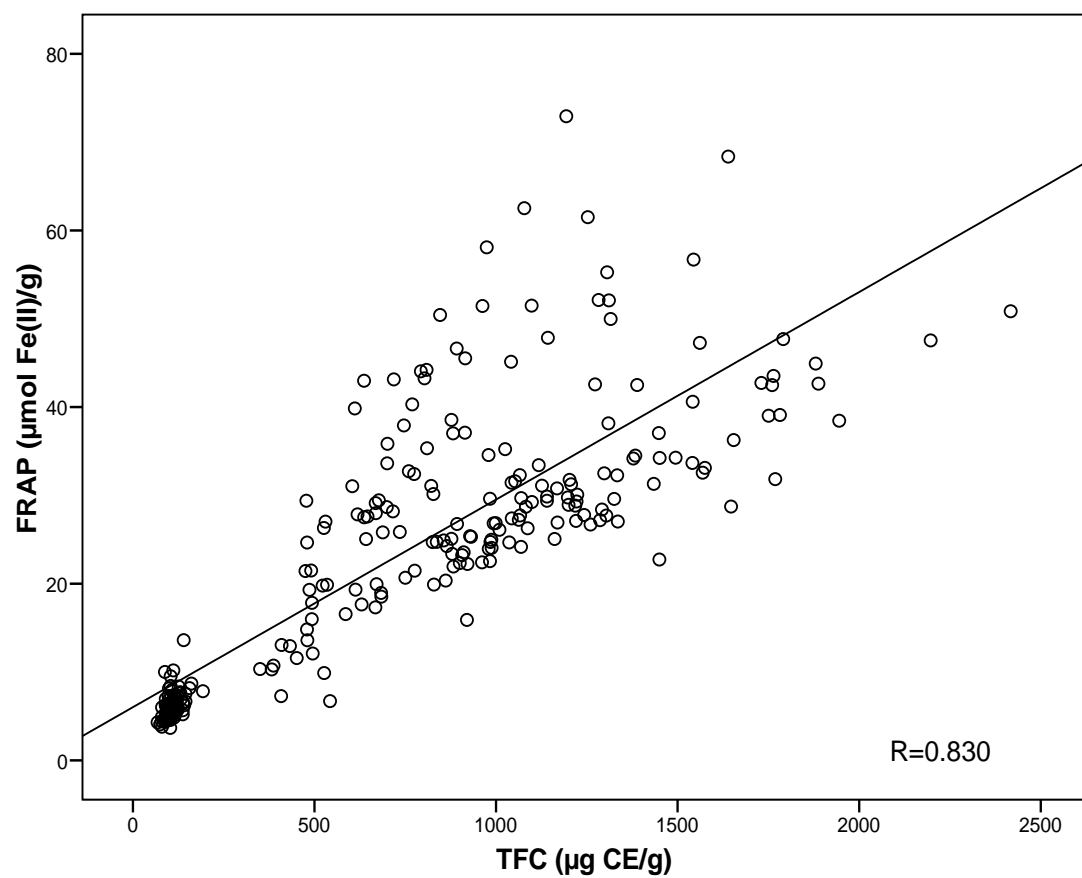

Figure S4. Correlation between TFC and FRAP.

Supplement: Supplementary file 1 [file foods-10-00864-s001.zip › Supplementary Figures 4.pdf]

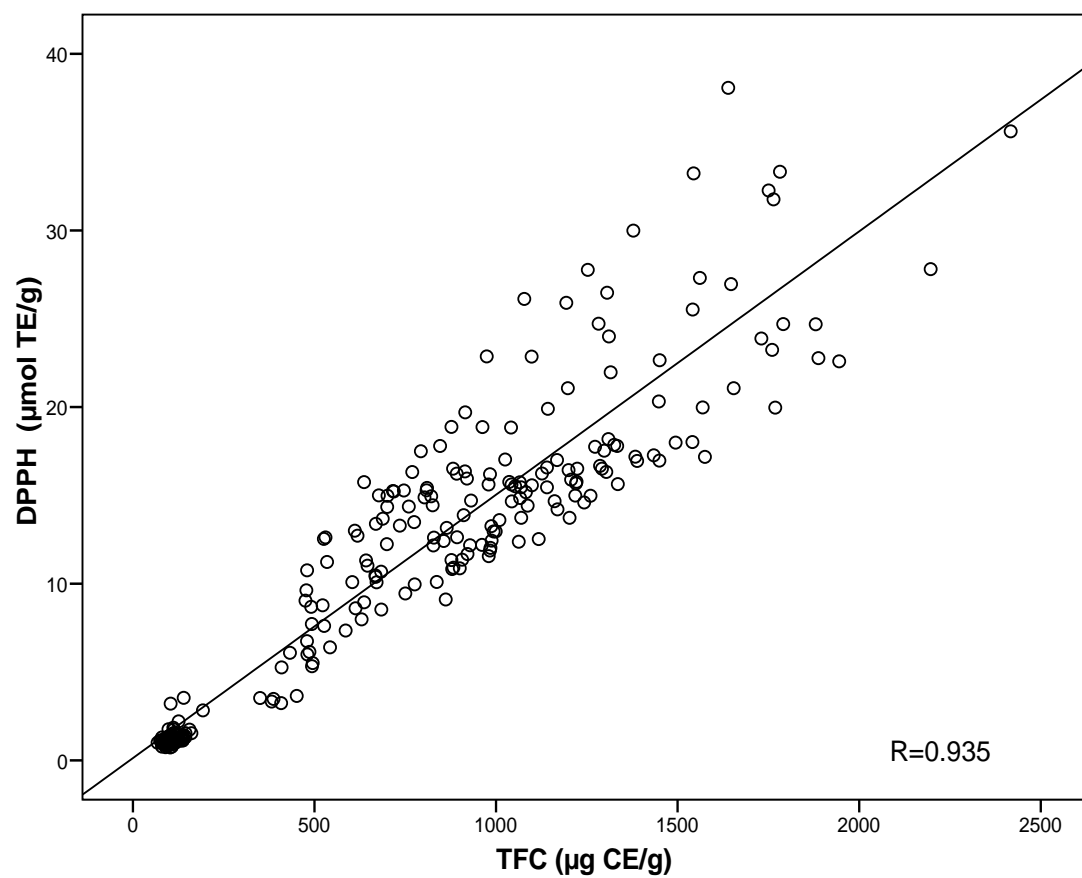

Figure S5. Correlation between TFC and DPPH.

Supplement: Supplementary file 1 [file foods-10-00864-s001.zip › Supplementary Figures 5.pdf]
